# DNA Barcoding in the Cycadales: Testing the Potential of Proposed Barcoding Markers for Species Identification of Cycads

**DOI:** 10.1371/journal.pone.0001154

**Published:** 2007-11-07

**Authors:** Chodon Sass, Damon P. Little, Dennis Wm. Stevenson, Chelsea D. Specht

**Affiliations:** 1 Department of Plant and Microbial Biology, University of California at Berkeley, Berkeley, California, United States of America; 2 Cullman Program for Molecular Systematics Studies, The New York Botanical Garden, Bronx, New York, United States of America; University of California at Davis, United States of America

## Abstract

Barcodes are short segments of DNA that can be used to uniquely identify an unknown specimen to species, particularly when diagnostic morphological features are absent. These sequences could offer a new forensic tool in plant and animal conservation—especially for endangered species such as members of the Cycadales. Ideally, barcodes could be used to positively identify illegally obtained material even in cases where diagnostic features have been purposefully removed or to release confiscated organisms into the proper breeding population. In order to be useful, a DNA barcode sequence must not only easily PCR amplify with universal or near-universal reaction conditions and primers, but also contain enough variation to generate unique identifiers at either the species or population levels. Chloroplast regions suggested by the Plant Working Group of the Consortium for the Barcode of Life (CBoL), and two alternatives, the chloroplast *psbA*-*trnH* intergenic spacer and the nuclear ribosomal internal transcribed spacer (nrITS), were tested for their utility in generating unique identifiers for members of the Cycadales. Ease of amplification and sequence generation with universal primers and reaction conditions was determined for each of the seven proposed markers. While none of the proposed markers provided unique identifiers for all species tested, nrITS showed the most promise in terms of variability, although sequencing difficulties remain a drawback. We suggest a workflow for DNA barcoding, including database generation and management, which will ultimately be necessary if we are to succeed in establishing a universal DNA barcode for plants.

## Introduction

Barcoding all described species is an enormous task with large sums being spent annually toward this end [1]. The proposed utility of the Barcode of Life project has been debated [2]–[8] and fundamental challenges have been acknowledged that focus on (a) the identification of DNA regions useful at the appropriate taxonomic level, (b) development of universal primers for these regions, and (c) the proper use of DNA barcodes as taxonomic identifiers. Proponents argue that molecular barcodes can be used to identify new species and eliminate the need for the complex taxonomic training that is currently required for species description and identification [9]—helping to ease the taxonomic crisis, especially in countries with high biodiversity and small numbers of practicing taxonomists. However the patterns of sequence variation make it logically impossible to use DNA barcodes for species circumscription as originally proposed [see 8 for an empirical example, see 10 for a theoretical example]. Although barcodes are appealing as a powerful tool to identify already described species, the cautious among us argue that the use of a single locus for identification may produce misleading results especially considering the different evolutionary histories of organellar and nuclear genomes within a single species [11]. Moreover, there is limited intraspecific sequence variation data for the proposed barcoding loci in plants. Others reject the use of barcodes for taxonomic purposes on the grounds that species description and identification requires full taxonomic revisions and that ‘phylogenies’ produced by barcoding genes do not necessarily represent evolutionary history [2], [12].

Ultimately the ability to identify a sample to species could be useful in cases where specimens are not of adequate quality to make accurate identifications (e.g. adult forms verses larval forms, sterile vouchers of plant specimens) and for ecologists and conservation biologists to rapidly assess biological diversity. In this sense, barcoding acts as a “forensic” tool for the accurate identification of a sample to species. The species, in this case, needs to be both described as unique (i.e. monographed) with a known range of morphological and sequence variation and be represented in a DNA barcoding database. This is an enormous task, requiring active participation of taxonomists, DNA sequencing facilities, database managers, and funding agencies to support monography, DNA sequencing, continuous specimen and database management, and potentially, the recircumscription of species as new data become available.

In order for a region of DNA to be operative as a barcode, it must simultaneously contain enough variability to be informative for identification (i.e. contain unique identifiers), be short enough to sequence in a single reaction, and contain invariant regions that can be used to develop universal primers [13]. Unfortunately, it is difficult to find a single region of DNA that has all three of these properties. For animals, the mitochondrial cytochrome oxidase I (*cox*I) gene has been successfully used for identification [5], [14], [15] although there are exceptions [for example], [see 8], [16,17]. For land plants, the *cox*I gene, and the mitochondrial genome in general, is not useful for identification at the species level because of low levels of primary sequence variability [18], [19]. Other regions often used for phylogenetic analysis across large groups of plants (e.g., *rbcL*) do not usually contain enough variability to identify individual species [but see 20], [21]. Developing a barcoding region for plants is further complicated by extensive genome-wide horizontal gene transfer, hybridization, and homoplasy [6].

Despite these obstacles, several gene regions have recently been proposed for use in land plants [4], [22], [23]. One set of loci includes a nuclear region, the ribosomal internal transcribed spacer with embedded 5.8S (nrITS), and a chloroplast region, the *psbA-trnH* intergenic spacer [4]. The combination of these two regions to positively and accurately identify taxa to species was tested on a subset of plants in the published analysis, but the combination is predicted to yield difficulties at the species level because nrITS is extremely variable in length—making analysis potentially more difficult—and *psbA-trnH* is likely to provide insufficient variation to reliably identify an organism to species, especially in groups with low divergence.

A portion of the chloroplast encoded large subunit ribosomal DNA, that is potentially “universally” amplifiable (Universial Plastid Aplicon; UPA), has also been proposed as a barcode for photosynthetic organisms. Available data suggest that although UPA may be variable at the species level in some algal lineages, it is not particularly variable among land plants [23].

**Figure 1:**
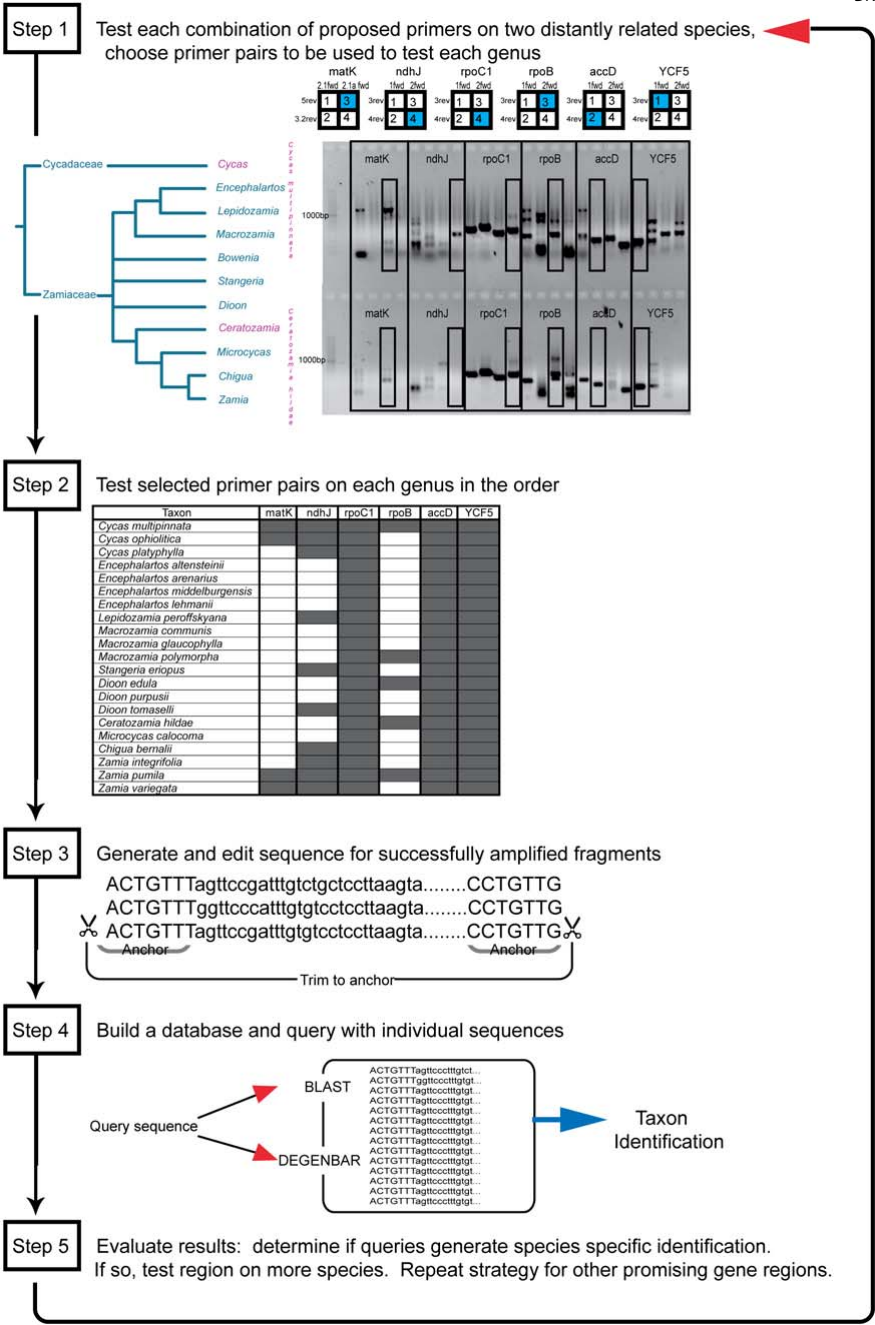
Barcoding optimization workflow. *Step 1*: genera used for testing all primer pairs, amplification products of each combination of primer pairs, and primer pair combination chosen for testing on more genera (highlighted in blue). *Step 2*: taxa subject to further testing and success of amplification with chosen primer pairs (highlighted in gray). *Step 3*: trimming all sequences to highly similar anchor regions. When possible, anchor regions were actually the primer binding sites. *Step 4*: each sequence entered into a database and used as a query sequence. The process is repeated for more species, with promising regions, or with new markers. The PWG suggested primer regions (www.kew.org/barcoding) are used as the example.

A consortium of institutions operating under the auspices of the Plant Working Group (PWG) of the Consortium for the Barcode of Life (CBoL) initially suggested five chloroplast gene regions for evaluation as potential barcodes: *matK*, *rpoC1*, *rpoB*, *accD* and *YCF5*, with *ndhJ* as a potential sixth region [http://www.kew.org/barcoding/]. These markers were proposed because of their potential for amplification with universal primers and because they may harbor sufficient sequence diversity, individually or in combination, to distinguish among species. In order for either of these criteria to be demonstrated, members of the community must devote time and effort to evaluating the proposed regions in the plant group they study with the goal of developing a defined “barcoding workflow” for the taxonomic group in question (Figure 1).

The last step of any barcoding workflow is to use newly generated sequence data in combination with a well-maintained database to positively identify the species in question. The BOLD identification system developed as part of the ongoing barcoding initiative at the University of Guelph (www.barcodinglife.org: [24]) uses a Hidden-Markov Model to align a query sequence to a reference database of *cox*I sequences generated for animal barcodes and then select the most similar sequence(s) as the identification. Unfortunately this algorithm is only applicable to sequences that can be globally aligned [24]. Some of the proposed plant barcodes are non-coding regions that cannot be sensibly aligned across land plants and thus could not benefit from BOLD-ID. Little and Stevenson [10] demonstrated that search algorithms can be successfully used on unaligned nucleotide sequence data, the most accurate and precise algorithms were, respectively, the commonly used local alignment search tool, BLAST [25] and a diagnostic method, DNA-BAR [26], [27]. DNA-BAR was originally intended as an algorithmic tool to select oligonucleotides for identification of microorganisms by Southern hybridization, but DNA-BAR's output file can be queried by a PERL script (DEGENBAR) that uses a simple matching algorithm to pick the most similar sequences(s) in the reference database. Provided that DNA-BAR is run on an input file containing each sequence and its reverse complement, both forward and reverse query sequences can be use to search the reference database.

The Cycadales are unique in their evolutionary position and importance for conservation, and as such are important to include in tests of proposed barcoding regions. Cycads are often thought of as “living fossils” and the extant taxonomic assemblage represents only a sampling of the ancient diversity. Most extant genera have representative fossils that date to the tertiary with some dating to the early Permian—indicating a minimum of 50-60 million years of morphological evolution that might enable us to observe greater nucleotide divergence than one would expect in more recently derived species [28]–[30]. Because of the relictual nature of the genera and their high value in illegal horticultural trade, cycads are an important focus for conservation efforts [31]. Most cycad genera are listed in CITES Appendix I and the remaining are listed in Appendix II [32]. An easy-to-use and inexpensive identification system would enable non-experts to identify illegally harvested individuals and help prevent the illegal trade of these species. Ideally it would be possible to identify an individual to species and perhaps even identify the population from whence the specimen was removed, allowing for proper repatriation of illegally harvested individuals.

The only way to determine if it is possible to use DNA barcodes across a wide variety of plant life is to test the proposed loci and search algorithms. In this study, we test the proposed barcoding regions in the members of the ancient gymnosperm order Cycadales in an effort to develop a functional barcoding workflow for this order.

## Results

### Proposed regions

The primer pairs chosen using *Ceratozamia hildae* and *Cycas ophiolitica* (Figure 1) for *ndhJ*, *rpoB* and *matK* did not work well for the remaining taxa (Table 1): non-specific primer binding resulted in multiple bands or complete lack of amplification. Because the purpose of these experiments was to test the functionality and utility of the proposed barcoding conditions and primers (as per www.kew.org/barcoding) on cycads we did not to develop novel cycad-specific primers or reaction conditions. Further analyses were performed only on those primers that successfully generated single products under universal conditions: *accD*, *YCF5* and *rpoC1*.

**Table 1:** Amplification success of suggested primer pairs (http://www.kew.org/barcoding/protocols.html) with broad sampling of cycads.

Marker	Successful amplification (single bands)	Non-specific amplification (multiple bands)	No amplification	Used for identification
*accD*	26/27 = 96%	1/27 = 4%	0/27 = 0%	yes
*YCF5*	66/66 = 100%	0/66 = 0%	0/66 = 0%	yes
*rpoC1*	29/29 = 100%	0/29 = 0%	0/29 = 0%	yes
*ndhJ*	12/21 = 57% *	6/21 = 29%	3/21 = 15%	no
*rpoB*	7/21 = 33%	14/21 = 67%	0/21 = 0%	no
*matK*	5/21 = 24%	11/21 = 52%	5/21 = 24%	no

Only markers with near universal amplification success were sequenced and tested for identification. An * indicates very weak bands.

**Figure 2:**
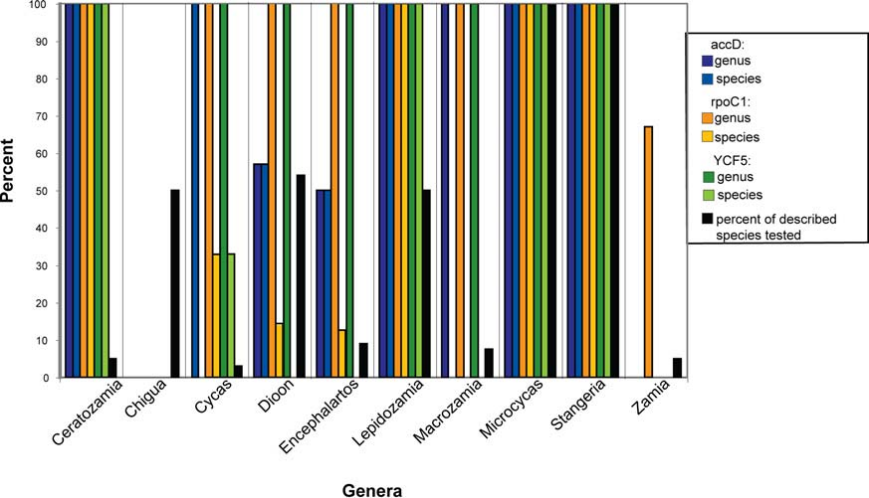
Success of species and genus level identification using CBoL proposed gene regions. Performance (% correct identification) at genus and species levels is noted for each marker for each of the 10 genera tested. No bar indicates failure of identification (0% success). Values are identical for BLAST and DNA-BAR/DEGENBAR.

Sequences generated from these three regions were tested for their ability to provide unique species identifications using both BLAST and DNA-BAR/DEGENBAR. Neither algorithm was able to positively identify individuals to species due to a lack of unique species-specific sequence for all species tested. Both algorithms had some success with identification of individuals to genus with 63–93% of query sequences correctly identified depending on the marker used (Figure 2). Inspection of the alignment revealed that there were very few variable positions. Over the three tested DNA regions, approximately 10% of the bases were variable (93 of 917 total bases): for *accD*, 28 of 242 base pairs were variable; for *rpoC1*, 41 of 476 base pairs were variable; and for *YCF5*, 24 of 199 base pairs were variable.

### Secondary regions (nrITS and *psb*A-*trn*H)

Because the chloroplast gene regions initially suggested by the Plant Working Group did not promise to distinguish among species even with our rather incomplete sampling, the alternative regions suggested by Kress et al. [4]–*psb*A-*trn*H intergenic spacer coupled with nrITS–were tested on the original 27–species set. The nrITS repeat (nrITS 1, 5.8S, and nrITS2) amplified cleanly in most species, but sequencing was difficult because nrITS in cycads (and other gymnosperms) is variable in length—approximately 1100 bp in most species, but around 1400 bp in *Stangeria eriopus*. In many species, the use of internal primers was required to generate contigs of the full sequence—making nrITS less desirable as a DNA barcode for cycads. A second potentially negative factor is the presence of long poly-G, poly-C, and poly-A repeats that are difficult to sequence through. Despite these issues, nrITS had sufficient variation to correctly identify individuals to species for the 27 individuals initially tested plus 4 additional species represented by sequences downloaded from GenBank (due to sequencing difficulties for *Zamia* and lack of fresh tissue samples for *Bowenia*).

Additional species were sampled from *Dioon* and *Encephalartos* to further test the functionality of nrITS for species-level identification. These genera were chosen because tissue samples were available that maximized the total percent coverage of species within each genus (7 out of 13 *Dioon*, 44 out of 65 *Encephalartos*). To further increase the number of species represented, available sequences from GenBank were included in the reference databases. For nrITS, a total of 96 sequences comprising 74 taxa were included in the ordinal-level analysis. Each genus was included in the ordinal analysis, and where possible more than one species from each genus was included (Table 2). In the ordinal-level database, all species were correctly identified to genus and 90.5% of queries correctly and uniquely identified the query sequence in the reference database. The success of self-identification is broken down by genus in Table 2. Genus–specific databases were made for *Encephalartos*, *Cycas*, and *Macrozamia* because some species could not be included in the ordinal–level database as the sequences did not contain the necessary anchor regions. In the generic-level databases the percent identification decreased: For *Encephalartos*, 26 of 44 (59.1%) species identified uniquely; for *Cycas*, 11 of 12 (91.7%) species identified uniquely; and for *Macrozamia,* 8 of 8 (100%) species identified uniquely. The nrITS locus had the highest success rate of the any of the markers tested; even though not all species could be correctly identified. Because of variation in length and sequence, complete alignments were not generated and the number of variable characters was not counted.

**Table 2:** nrITS identification success for each genus.

Genera	Number of species analyzed		Total number of named species [40]	Percent of species that are represented and success rate of unique identification in ordinal level and generic level analyses			
	Order level	Genus level		Ordinal level		Generic level	
				Percent represented	Success	Percent represented	Success
*Cycas*	11	12	99	11.1	11/11 = 100%	12.1	11/12 = 91.7%
*Zamia*	8	–	59	13.6	8/8 = 100%	–	–
*Chigua*	2	–	2	100	2/2 = 100%	–	–
*Ceratozamia*	8	–	21	38.1	8/8 = 100%	–	–
*Macrozamia*	7	8	40	17.5	7/7 = 100%	20	8/8 = 100%
*Stangeria*	1	–	1	100	1/1 = 100%	–	–
*Encephalartos*	25	44	65	38.5	18/25 = 72%	67.7	26/44 = 59.1%
*Lepidozamia*	2	–	2	100	2/2 = 100%	–	–
*Microcycas*	1	–	1	100	1/1 = 100%	–	–
*Bowenia*	2	–	2	100	2/2 = 100%	–	–
*Dioon*	7	–	13	53.8	7/7 = 100%	–	–
**TOTAL**	**74**	**94**	**305**	**24.2**	**67/74 = 90.1%**	**32.5**	**76/94 = 80.9%**

Success indicates results for both BLAST and DNA-BAR/DEGENBAR, which were identical.

**Figure 3:**
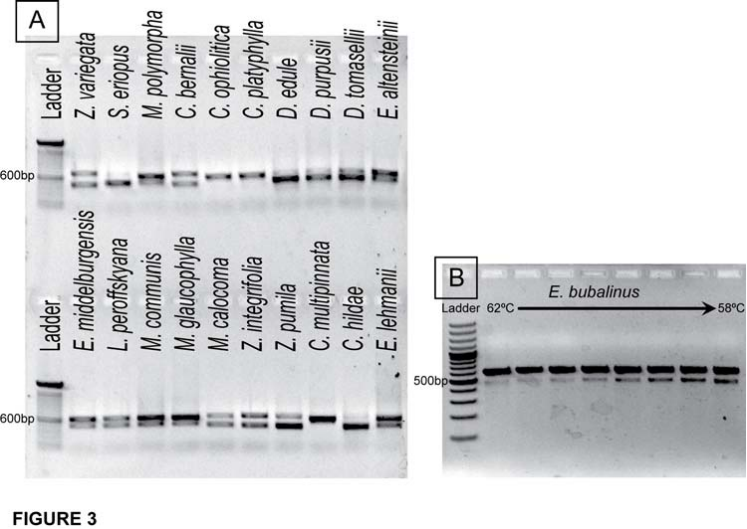
Amplification of PCR producing using published *psbA-trnH* primers. A: All genera except Cycas showed double bands, some genera had more prominent smaller fragments (e.g. *Dioon*), while others had more prominent larger fragments (e.g. *Macrozamia*). B: When more stringent reaction conditions were applied by running the amplification with the primer annealing temperature at 62°C, double bands were still evident.

The *psb*A-*trn*H spacer primers and reaction conditions specified by Kress et al. [4] yielded distinct double bands in all but *Cycas* species (Figure 3A). Even with greatly increased annealing temperature, double bands were still present (Figure 3B). The utility of this region for barcoding was tested by sequencing the larger of the two fragments (after gel excision) from species that could not be uniquely identified in the nrITS database. The addition of *psb*A-*trn*H sequence data did not further resolve the non-specific identifications made by nrITS for the species tested (Table S1). Of 322 total characters, including gaps, in the cursory alignment used to ensure the presence of anchor regions, 83 characters were parsimony informative; this variability was mostly due to differences between *Cycas* and the remaining genera and is not directly translated into sequence variation that is useful for barcoding.

### Algorithm differences: BLAST and DNA-BAR/DEGENBAR

As tested on our cycad database, there were no differences in the ability of BLAST and DNA-BAR/DEGENBAR to correctly identify species.

## Discussion

### Proposed barcoding loci

Three of the 6 regions proposed by the Plant Working Group did not easily amplify across all Cycadales. A recently posted Phase II update based on research from the Plant Working Group (http://www.kew.org/barcoding/update.html) indicates a new primer pair for *matK* that was successful in *Encephalartos* and may be successful across all genera of cycads, while *YCF5* was determined to not be suitable as a barcode region for all land plants due to its apparent absence in bryophytes. In addition, the Phase II update suggests two options for a combined approach to DNA barcoding, involving the use of three gene regions to accurately identify a sample to species. Option one uses *rpoC1*, *rboB* and *matK* while option two utilizes *rpoC1*, *matK* and *trnH-psbA*. However, neither of these options are likely to provide the resolution necessary to identify a cycad sample to species based on the results presented above. The three regions that did easily amplify and were tested (including *rpoC1*, a member of both proposed three-region barcode options) did not provide enough variation to specifically identify the cycads tested in this study. It is possible that *matK* may provide more variation than other regions tested in this analysis, and continued studies with the newly proposed primers are necessary to evaluate its utility as a barcoding region for cycads. Our results are emblematic of the challenge faced in plant DNA barcoding. Additional search is required to find regions that both amplify easily and contain variation if the goal of a universal primer set (or sets) is to be reached.

### nrITS

nrITS shows promise as a barcoding region because it contains enough variability to identify many samples to species. However, intraspecific differences are greater than interspecific differences in some cases. For the 14 species for which multiple individuals were sampled, 8 of the BLAST queries resulted in correct species identification, but the next highest BLAST hit (based on e-value) was not the correct species (data not shown). Many differences between species can be attributed to only one base pair difference. Six of the 26 unique identifications within *Encephalartos* were only a one base pair different from the next highest BLAST hit, and 17 of the 26 had less than 5 base pair differences. This suggests that once all species of *Encephalartos* have been sampled there may be a complete lack of informative variation. In addition, because sequences are generated directly from amplifications of whole genomic DNA, rare alleles (less than 10% of the amplicon) may not be evident and potential variation within a species will be missed. If nrITS is to be used as barcoding locus, further sequencing using cloned PCR products will be necessary to ensure that all alleles of each species are captured in the database. Allelic variation could result in false identification if all alleles for each species are not included in the reference database [33]. Finally, because some identifications are based on single nucleotide positions, sequencing errors could cause further false identifications.

### psbA-trnH

The placement of *psbA* has shifted in and out of the chloroplast inverted repeat in various lineages making the *psbA-trnH* intergenic spacer difficult to work with. For example, in ferns *psbA* is located inside the inverted repeat [34]; in eudicots it is located outside of the repeated region; in *Pinus contorta*, *psbA* has undergone a tandem duplication, with one truncated copy [35], [36]. In cycads, PCR with the *psbA-trnH* primers suggested by Kress et al.[4] generated two products in all genera tested except *Cycas* (Figure 3). Additional primers designed to include more of the *psb*A coding region and a portion of *psb*A-*trn*H intergenic spacer specific to either the small or large fragment (Table 3) were tested on *Encephalartos nubimontanus*. Sequences from these amplifications had two distinct protein coding regions, indicating that *psbA* is present in two copies in some cycads or is present as a pseudogene. The longer fragment in *Encephalartos nubimontanous* corresponded to the protein sequence that is most similar to *psbA* protein sequence of other gymnosperms (*Ginkgo biloba* and *Pinus korianus*). These analyses were performed on whole genomic DNA, so it remains unclear whether both genes are being amplified from the chloroplast genome or if the second fragment could be nuclear DNA that was transferred from the chloroplast, a well documented phenomenon [37]. Problems with amplification aside, *psbA-trnH* does not show promise as a barcoding locus for cycads because of its inability to provide specific identification for taxa that could not be distinguished with nrITS (Table S1).

**Table 3:** Primers and reaction conditions used in this study.

Gene	Primer	Sequence 5′-3′	Rxn conditions	
*matK*	2.1 forward	CCTATCCATCTGGAAATCTTAG	94°C–4 min	
	2.1a forward	ATCCATCTGGAAATCTTAGTTC	94°C-30 sec	
	5 reverse	GTTCTAGCACAAGAAAGTCG	53°C-40 sec	40×
	3.2 reverse	CTTCCTCTGTAAAGAATTC	72°C-40 sec	
			72°C-7 min	
*ndhJ* (1st reserve)	1 forward	CATAGATCTTTGGGCTTYGA	94°C–4 min	
	2 forward	TTGGGCTTCGATTACCAAGG	94°C-30 sec	
	3 reverse	ATAATCCTTACGTAAGGGCC	53°C-40 sec	40×
	4 reverse	TCAATGAGCATCTTGTATTTC	72°C-40 sec	
			72°C-7 min	
*rpoC1*	1 forward	GTGGATACACTTCTTGATAATGG	94°C–4 min	
	2 forward	GGCAAAGAGGGAAGATTTCG	94°C-30 sec	
	3 reverse	TGAGAAAACATAAGTAAACGGGC	53°C-40 sec	40×
	4 reverse	CCATAAGCATATCTTGAGTTGG	72°C-40 sec	
			72°C-7 min	
*rpoB*	1 forward	AAGTGCATTGTTGGAACTGG	94°C–4 min	
	2 forward	ATGCAACGTCAAGCAGTTCC	94°C-30 sec	
	3 reverse	CCGTATGTGAAAAGAAGTATA	53°C-40 sec	40×
	4 reverse	GATCCCAGCATCACAATTCC	72°C-40 sec	
			72°C-7 min	
*accD*	1 forward	AGTATGGGATCCGTAGTAGG	94°C–4 min	
	2 forward	GGRGCACGTATGCAAGAAGG	94°C-30 sec	
	3 reverse	TTTAAAGGATTACGTGGTAC	53°C-40 sec	40×
	4 reverse	TCTTTTACCCGCAAATGCAAT	72°C-40 sec	
			72°C-7 min	
*YCF5*	1 forward	GGATTATTAGTCACTCGTTGG	94°C–4 min	
	2 forward	ACTTTAGAGCATATATTAACTC	94°C-30 sec	
	3 reverse	ACTTACGTGCATCATTAACCA	53°C-40 sec	40×
	4 reverse	CCCAATACCATCATACTTAC	72°C-40 sec	
			72°C-7 min	
*psbA-trnH* [4]	fwd	GTTATGCATGAACGTAATGCTC	94°C–5 min	
	rev	CGCGCATGGTGGATTCACAATCC	94°C-1 min	
			55°C-1 min	30×
			72°C-1.5 min	
			72°C-7 min	
*psbA-trnH* (including protein coding region)	fwd	CGAGCCTGTTTCTGGTTCTC	98°C–3 min	
	Rev (short-fragment)	GGGGTGTGGGTAGAGCAGT	98°C-10 sec	
			60°C [−0.5°/cycle] −20 sec	10×
	Rev (long-fragment)	CCGACGACGAACTAACATTTG	72°C-1 min	
			98°C-10 sec	
			55°C-20 sec	25×
			72°C-1 min	
			72°C-7 min	
nrITS	5a fwd	CCTTATCATTTAGAGGAAGGAG	94°C–5 min	
	4 rev	TCCTCCGCTTATTGATATGC	94°C-1 min	
			50°C-1 min	30×
			72°C-1.5 min +3 sec /cycle	
			72°C-7 min	
	2c rev (sequencing only)	GCTACGTTCTTCATCGTGGC	N/A	

Conditions for chloroplast markers from the Plant Working Group (www.kew.org/barcoding/protocols.html); conditions for *psbA-trnH* adopted from Kress et al. 2005.

### Algorithm comparisons: BLAST and DNA-BAR/DEGENBAR

For our data sets, there was no difference between BLAST and DNA-BAR/DEGENBAR. For optimization, BLAST offers several advantages: It generates a more detailed output and is readily available and downloadable from NCBI. For use in barcoding in practice, either method seems to be similarly successful [10]. Standardization of an algorithm used for database searches as part of the DNA barcoding workflow should be promoted in order to provide maximally consistent results.

### Conclusions

The goal of finding universal primer pairs and reaction conditions with unique internal sequence for all land plants remains elusive—not surprising given the complex history of land plant genomes. At least in cycads, the chloroplast regions tested do not have sufficient variability to provide the unique sequences (characters or combinations of characters) necessary to identify an individual to species. Nuclear regions may provide more usable variability, but such regions have not yet been identified. Perhaps a set of primers designed for each of the major clades of land plants (such as gymnosperms, pteridophytes, angiosperms, mosses, etc.) could be used simultaneously if universal tails were added to the primers so that although only one set of primers would amplify an unknown sample, the amplicon could be sequenced using a primer that matched the tail sequence. This approach would be especially useful in situations where little morphological information is available from the sample (e.g., determination of diet based on scat collections, identification of degraded, fragmented or sterile tissue). Alternatively improved technology such as sequencing long regions of DNA (e.g., whole or partial chloroplast genomes) may enable identification based on both genome architecture and additional variation captured by simply increasing the total amount of sequence.

## Materials and Methods

### Taxon sampling and primer testing strategy

For each region, the Plant Working Group designed 4 primers (2 forward and 2 reverse) in their Phase I trials (www.kew.org/barcoding) in an attempt to increase the likelihood of finding a working combination. The primer pairs were first tested in all combinations on two species—*Cycas ophiolitica* and *Ceratozamia hildae*—chosen based on their distant placement in the Cycadales phylogeny. Primers were considered successful if they amplified a single product. If a single band was obtained by more than one primer pair, the pair that generated the largest and brightest (highest PCR yield) of the bands was chosen. If amplification was successful in only one of the two species, the pair generating the brightest band for that species was selected. The best working primers were then tested for a set of 21 species representing 10 of 11 cycad genera. Gene regions with universal or near universal success in amplification were sequenced. Gene regions with variability that enabled specific positive identification were tested on additional species within each genus to further test the region's ability to provide identification at the species level. This workflow is outlined in Figure 1.

### Plant collection, DNA extraction, and amplification

Leaflets were clipped from live plants, dried in silica gel, and then stored at −80°C. Whole genomic DNA was extracted using DNeasy Plant Mini Kits (Qiagen, Valencia, CA) or a modified CTAB method [38] from fresh or frozen tissue. PCR amplification was performed from genomic DNA according to instructions on Kew's website (www.kew.org/barcoding) for the 6 chloroplast regions or following Kress et al. [4] (Table 3). Some modifications were made to accommodate the use of iProof™ High-Fidelity DNA polymerase (Bio-Rad, Hercules, CA). Amplified products were inspected on 1% agarose/TAE gels. Amplicon was cleaned by digestion with Exonuclease and Shrimp alkaline phosphatase or through gel extraction using the QiaQuick™ Gel Extraction Kit (Qiagen, Valencia, CA). Cycle sequencing was performed using Amplitaq™ (Amersham, Piscataway, NJ) or BigDye® v3.1 (Applied Biosystems, Foster City, CA) sequencing chemistry and an ABI PRISM® 3100 sequencer (Applied Biosystems, Foster City, CA).

### Sequence alignment and determination of barcoding regions

Sequence editing and contig generation were performed using Sequencher (Gene Codes Corp., Ann Arbor, MI). Additional sequences for nrITS were downloaded from GenBank (see Table S1). If sequences did not include primer regions, all sequences were trimmed to an area with highly similar (>98% identity) sequence regions at the ends of the sequence reads—anchor regions (Figure 1). This was only necessary for nrITS and *psb*A sequences. Sequences from these loci were longer and more variable than other regions and as a result primer regions were not always sequenced. Sequences were used for further analysis only if they contained the anchor regions—ensuring that identification success was due to internal variability and not arbitrary factors such as sequence read length. In order to identify and trim sequences to the anchor regions, nrITS and *psb*A-*trn*H regions were aligned using CLUSTAL W [39] and then manually adjusted using MacClade (Sinauer Associates, Inc., Sunderland, MA). After elimination of sequences with ambiguous nucleotides and non-anchor containing sequences, databases were created and individual sequences were queried against the databases with BLAST and DNA-BAR/DEGENBAR. Sequences excluded from the ordinal level database due to the absence of anchor regions were included in secondary databases that contained only a single genus provided that genus-specific anchors could be identified from sequences that were not long enough to be included in the ordinal-level database (this was only the case for nrITS sequences from *Encephalartos*, *Cycas* and *Macrozamia*; Table 2).

### Comparability of results with different algorithms: BLAST and DNA-BAR/DEGENBAR

The same sets of sequences were used both to generate databases and as query sequences for both BLAST and DNA-BAR/DEGENBAR [10]. BLAST queries were run without filtering. Before generating the database with DNA-BAR the sequences were run through a PERL script that added a reverse complement for each sequence in order to ensure that query sequences would match the database in either the forward or the reverse orientation. To test for unique species-specific barcodes that could be used for a species level identification, the sequence belonging to each species was copied from the database and used as a query sequence. If the query sequence returned an exact match only to itself, this was scored as a positive identification at the species level. If the query sequence returned an exact match to itself and other members of the same genus, this was scored as a negative identification at the species level, but a positive identification to the genus level. DNA-BAR/DEGENBAR returns only the highest scoring match(es), so the cutoff for genus and species identification is straightforward. For BLAST, an additional constraint was added: to positively score an identification at the genus level the best match as well as the next most similar sequence had to match the genus of the query sequence. If any other genus was included in the top two hits, the result was not considered genus specific.

## Supporting Information

Table S1 Supplemental Data: List of GenBank ID numbers of taxa used in tests for barcoding utility. Numbers in bold showed unique identifications in both BLAST and DNA-BAR/DEGENBAR, numbers in plain text were not identified uniquely. Results shown are those from the database with the most inclusive species sampling (i.e. the genus level database in the case of *Encephalartos*, *Cycas* and *Macrozamia* for nrITS).
(0.33 MB DOC)
