# Supplementary material for: DNA Barcoding in the Cycadales: Testing the Potential of Proposed Barcoding Markers for Species Identification of Cycads
Source: PLoS One. 2007 Nov 7;2(11):e1154. doi: 10.1371/journal.pone.0001154 (PMC2063462; doi:10.1371/journal.pone.0001154)
Supplement: Table S1 — Supplemental Data: List of GenBank ID numbers of taxa used in tests for barcoding utility. Numbers in bold showed unique identifications in both BLAST and DNA-BAR/DEGENBAR, numbers in plain text were not identified uniquely. Results shown are those from the database with the most inclusive species sampling (i.e. the genus level database in the case of Encephalartos, Cycas and Macrozamia for nrITS). (0.33 MB DOC) [file pone.0001154.s001.doc]

**Table S1. List of GenBank ID numbers of taxa used in tests for barcoding utility**

|  | **Marker** | | | | | |
| --- | --- | --- | --- | --- | --- | --- |
| **Taxa** | nrITS | *psbA-trnH* | *accD* | *YCF5* | *rpoC1* | Reference/ Accession |
| *Bowenia serrulata* | **AB076190** | -- | -- | -- | -- | [41] |
| *Bowenia spectabilis* | **AF531228** | -- | -- | -- | -- | [42] |
| *Bowenia spectabilis* | **AB076191** | -- | -- | -- | -- | [41] |
| *Ceratozamia brevifrons* | **AF407304** | -- | -- | -- | -- | [43] |
| *Ceratozamia hildae* | **EF612912** | -- | **EF612896** | **EF612824** | **EF612803** | UCBG 2005.1112 |
| *Ceratozamia kuesteriana* | **AB076239** | -- | -- | -- | -- | [41] |
| *Ceratozamia mexicana* | **AB076196** | -- | -- | -- | -- | [41] |
| *Ceratozamia mexicana var. robusta* | **AF407290** | -- | -- | -- | -- | [43] |
| *Ceratozamia miqueliana* | **AF531240** | -- | -- | -- | -- | [42] |
| *Ceratozamia norstogii* | **AF531241** | -- | -- | -- | -- | [42] |
| *Ceratozamia robusta* | **AB076197** | -- | -- | -- | -- | [41] |
| *Ceratozamia zaragozae* | **AF407302** | -- | -- | -- | -- | [43] |
| *Chigua bernalii* | **EF612913** | -- | EF612907 | EF612823 | EF612818 | UCBG 2005.1116 |
| *Chigua restrepoi* | **AB076199** | -- | -- | -- | -- | [41] |
| *Chigua restrepoi* | **AF531243** | -- | -- | -- | -- | [42] |
| *Cycas circinalis* | **AF531222** | -- | -- | -- | -- | [42] |
| *Cycas furfuracea* | **AF531225** | -- | -- | -- | -- | [42] |
| *Cycas micholitzii* | **AB076240** | -- | -- | -- | -- | [41] |
| *Cycas multipinnata* | **AB076202** | -- | -- | -- | -- | [41] |
| *Cycas multipinnata* | **EF612914** | **EF612963** | EF612898 | **EF612825** | **EF612807** | UCBG KH 220 |
| *Cycas ophiolitica* | **EF612915**, EF612916 | EF612962 | EF612899 | EF612826 | EF612810 | UCBG 2004.0917 |
| *Cycas pectinata* | **AB076204** | -- | -- | -- | -- | [41] |
| *Cycas platyphylla* | EF653157 | EF612961 | EF612895 | EF612827 | EF612804 | UCBG 2004.0923 |
| *Cycas revoluta* | **AF531223** | -- | -- | -- | -- | [42] |
| *Cycas revoluta* | **X91870** | -- | -- | -- | -- | [44] |
| *Cycas rumphii* | **AB076205** | -- | -- | -- | -- | [41] |
| *Cycas taitungensis* | **AB076188** | -- | -- | -- | -- | [41] |
| *Cycas thouarsii* | **AB076206** | -- | -- | -- | -- | [41] |
| *Dioon edule* | **AF531229** | -- | -- | -- | -- | [42] |
| *Dioon edule* | **AB076208** | -- | -- | -- | -- | [41] |
| *Dioon edule* | **EF612917, EF612918** | -- | EF612911 | EF612829 | EF612808 | UCBG 2004.0985 |
| *Dioon califanoi* | **EF612919** | -- | **EF612892** | EF612828 | **EF612796** | UCBG 90.1177 |
| *Dioon mejiae* | **EF612920** | -- | **EF612887** | EF612830 | EF612799 | UCBG 2004.0993 |
| *Dioon purpusii* | **EF612921** | -- | EF612909 | EF612831 | EF612809 | UCBG 2004.1010 |
| *Dioon sonorense* | **EF612922** | -- | **EF612889** | EF612832 | EF612797 | UCBG 2004.1346 |
| *Dioon spinulosum* | **AB076209** | -- | -- | -- | -- | [41] |
| *Dioon spinulosum* | -- | -- | **EF612886** | EF612833 | EF612798 | UCBG 2004.0995 |
| *Dioon tomasellii* | **AB076211** | -- | -- | -- | -- | [41] |
| *Dioon tomasellii* | **AF531230** | -- | -- | -- | -- | [42] |
| *Dioon tomasellii* | -- | -- | EF612908 | EF612834 | EF612802 | UCBG 2004.0998 |
| *Encephalartos altensteinii* | **EF612923, EF612924** | **EF612952** | EF612893 | EF612835 | EF612822 | UCBG 2005.0782 |
| *Encephalartos middelburgensis* | EF612925 | EF612953 | EF612910 | EF612859 | EF612821 | UCBG 2005.0853 |
| *Encephalartos arenarius* | EF612926 | EF653162 | EF612894 | EF612836 | EF612820 | UCBG 2005.0965 |
| *Encephalartos arenarius* | **AF531237** | -- | -- | -- | -- | [42] |
| *Encephalartos bubalinus* | **EF612927** | -- | **EF612885** | EF612837 | EF612794 | UCBG 94.0857 |
| *Encephalartos caffer* | **EF612928** | -- | **EF612890** | EF612838 | EF612795 | UCBG 2005.0948 |
| *Encephalartos concinnus* | **EF612929** | -- | -- | EF612839 | EF612800 | UCBG 2005.0917 |
| *Encephalartos cupidus* | EF612930 | EF653160 | -- | EF612840 | **EF612801** | UCBG 2005.0997 |
| *Encephalartos cycadifolius* | **AB076214** | -- | -- | -- | -- | [41] |
| *Encephalartos dolomiticus* | EF653139 | -- | -- | EF612841 | -- | UCBG 2005.0866 |
| *Encephalartos dyerianus* | EF653140 | EF653161 | -- | EF612842 | -- | UCBG 2005.0867 |
| *Encephalartos eugene-maraisii* | EF653141 | -- | -- | EF612843 | -- | UCBG 2005.1057 |
| *Encephalartos ferox* | **EF653142** | -- | -- | EF612844 | -- | UCBG 66.1511 |
| *Encephalartos friderici-guilielmi* | **EF653143** | -- | -- | EF612845 | -- | UCBG 93.0490 |
| *Encephalartos ghellinckii* | -- | -- | -- | EF612846 | -- | UCBG 94.0878 |
| *Encephalartos ghellinckii* | **AF531236** | -- | -- | -- | -- | [42] |
| *Encephalartos gratus* | **AB076216** | -- | -- | -- | -- | [41] |
| *Encephalartos gratus* | -- | -- | -- | EF612847 | -- | UCBG 74.0217 |
| *Encephalartos hildebrandtii* | **EF612931** | -- | -- | EF612848 | -- | UCBG 90.1509 |
| *Encephalartos hirsutus* | **EF612932** | -- | -- | EF612849 | -- | UCBG 2005.0170 |
| *Encephalartos horridus* | EF653144 | EF653164 | -- | EF612850 | -- | UCBG 2005.1002 |
| *Encephalartos horridus x longifolius* | EF653145 | -- | -- | EF612851 | -- | UCBG 2005.0404 |
| *Encephalartos humilus* | EF653146 | -- | -- | EF612852 | -- | UCBG 2005.0844 |
| *Encephalartos inopinus* | **EF653147** | -- | -- | EF612853 | -- | UCBG 2005.0932 |
| *Encephalartos lanatus* | **AB076217** | -- | -- | -- | -- | [41] |
| *Encephalartos lanatus* | **EF653148** | -- | -- | EF612854 | -- | UCBG 2005.0745 |
| *Encephalartos laurentianus* | EF653149 | -- | -- | **EF612855** | -- | UCBG 2005.1155 |
| *Encephalartos laevifolius* | EF612933 | -- | -- | -- | -- | UCBG 2005.0862 |
| *Encephalartos lebomboensis* | EF612934 | EF612955 | -- | EF612856 | -- | UCBG 2005.0908 |
| *Encephalartos lehmanii* | EF612935 | EF612954 | **EF612891** | -- | EF612819 | UCBG 2005.1022 |
| *Encephalartos longifolius* | **AB076219** | -- | -- | -- | -- | [41] |
| *Encephalartos longifolius* | -- | -- | -- | EF612857 | -- | UCBG 2005.0893 |
| *Encephalartos macrostrobilus* | **EF612936** | -- | -- | EF612858 | -- | UCBG 2005.1089 |
| *Encephalartos macrostrobilus* | EF612937 | EF653159 | -- | -- | -- | UCBG 2005.1060 |
| *Encephalartos natalensis* | EF612938 | -- | -- | EF612860 | -- | UCBG 68.0769 |
| *Encephalartos nubimontanus* | EF612939 | **EF612958** | -- | EF612861 | -- | UCBG 2005.0868 |
| *Encephalartos paucidentatus* | **EF612940** | -- | -- | EF612862 | -- | UCBG 2005.0848 |
| *Encephalartos princeps* | EF612941 | **EF653163** | -- | EF612863 | -- | UCBG 2005.1067 |
| *Encephalartos rosiensis* | **EF653150** | -- | -- | EF612864 | -- | UCBG 67.0423 |
| *Encephalartos schaijesii* | **EF653151** | -- | -- | **EF612865** | -- | UCBG 2005.0907 |
| *Encephalartos schmitzii* | **EF653152** | -- | -- | EF612866 | -- | UCBG 2005.1026 |
| *Encephalartos sclavoi* | EF653153 | -- | -- | EF612867 | -- | UCBG 2005.0001 |
| *Encephalartos senticosus* | EF653154 | -- | -- | EF612868 | -- | UCBG 2005.1020 |
| *Encephalartos sp* | EF612942 | -- | -- | -- | -- | UCBG 2005.0903 |
| *Encephalartos tegulaneus* | **EF653155** | -- | -- | EF612869 | -- | UCBG 96.0179 |
| *Encephalartos transvenosus* | **EF612943** | -- | -- | EF612870 | -- | UCBG 2005.0930 |
| *Encephalartos trispinosus* | EF612944 | **EF612956** | -- | EF612871 | -- | UCBG 2005.1013 |
| *Encephalartos turnerii* | **EF653156** | -- | -- | EF612872 | -- | UCBG 2005.0977 |
| *Encephalartos umbeluziensis* | **EF612945** | -- | -- | EF612873 | -- | UCBG 2005.0806 |
| *Encephalartos villosus* | EF612946 | EF612957 | -- | EF612874 | -- | UCBG 2005.0787 |
| *Encephalartos whitelockii* | **EF612947** | -- | -- | EF612875 | -- | UCBG 2005.1055 |
| *Lepidozamia peroffskyana* | **EF612948** | -- | **EF612901** | **EF612876** | **EF612813** | UCBG 71.0346 |
| *Lepidozamia hopei* | **AB076222** | -- | -- | -- | -- | [41] |
| *Lepidozamia hopei* | **AF531238** | -- | -- | -- | -- | [42] |
| *Lepidozamia peroffskyana* | **AB076225** | -- | -- | -- | -- | [41] |
| *Lepidozamia peroffskyana* | **AF531239** | -- | -- | -- | -- | [42] |
| *Macrozamia communis* | **AB076241** | -- | -- | -- | -- | [41] |
| *Macrozamia communis* | **AF531232** | -- | -- | -- | -- | [42] |
| *Macrozamia communis* | **EF653158** | -- | EF612903 | EF612878 | EF612815 | UCBG 2004.0930 |
| *Macrozamia elegans* | **AF531233** | -- | -- | -- | -- | [42] |
| *Macrozamia fraseri* | **AF531235** | -- | -- | -- | -- | [42] |
| *Macrozamia glaucophylla* | **EF612949** | EF612959 | EF612902 | EF612879 | EF612814 | UCBG 2004.0943 |
| *Macrozamia lucida* | **AB076226** | -- | -- | -- | -- | [41] |
| *Macrozamia moorei* | **AB076227** | -- | -- | -- | -- | [41] |
| *Macrozamia moorei* | **AF531231** | -- | -- | -- | -- | [42] |
| *Macrozamia pauli-guilielmi* | **AF531234** | -- | -- | -- | -- | [42] |
| *Macrozamia polymorpha* | **EF612950** | EF612960 | EF612900 | EF612880 | EF612812 | UCBG 2004.0971 |
| *Microcycas calocoma* | -- | -- | **EF612888** | **EF612877** | **EF612811** | UCBG 2005.0695 |
| *Microcycas calocoma* | **AB076229** | -- | -- | -- | -- | [41] |
| *Microcycas calocoma* | **AF531242** | -- | -- | -- | -- | [42] |
| *Stangeria eriopus* | **AF531226** | -- | -- | -- | -- | [42] |
| *Stangeria eriopus* | **AB076242** | -- | -- | -- | -- | [41] |
| *Stangeria eriopus* | **EF612951** | -- | **EF612897** | **EF612881** | **EF612806** | UCBG 58.1003 |
| *Zamia angustifolia* | **AB076243** | -- | -- | -- | -- | [41] |
| *Zamia fairchildiana* | **AB076232** | -- | -- | -- | -- | [41] |
| *Zamia fischeri* | **AB076234** | -- | -- | -- | -- | [41] |
| *Zamia integrifolia* | **AB076244** | -- | -- | -- | -- | [41] |
| *Zamia integrifolia* | -- | -- | EF612906 | EF612882 | EF612817 | UCBG 2005.1132 |
| *Zamia floridana* | **AF531244** | -- | -- | -- | -- | [42] |
| *Zamia lindenii* | **AF531245** | -- | -- | -- | -- | [42] |
| *Zamia pumila* | **AB076237** | -- | -- | -- | -- | [41] |
| *Zamia pumila* | -- | -- | EF612905 | EF612883 | EF612816 | UCBG 61.1036 |
| *Zamia skinneri* | **AF531246** | -- | -- | -- | -- | [42] |
| *Zamia variegata* | -- | -- | EF612904 | EF612884 | EF612805 | UCBG 2005.1149 |

Numbers in bold showed unique identifications in both BLAST and DNA-BAR/DEGENBAR, numbers in plain text were not identified uniquely. Results shown are those from the database with the most inclusive species sampling (i.e. the genus-level database in the case of *Encephalartos*, *Cycas* and *Macrozamia* for nrITS).
